# Nocardiosis in Solid Organ Transplant Recipients: 10-Year Single Center Experience and Review of Literature

**DOI:** 10.3390/microorganisms12061156

**Published:** 2024-06-06

**Authors:** Julia Bini Viotti, Jacques Simkins, John M. Reynolds, Gaetano Ciancio, Giselle Guerra, Lilian Abbo, Shweta Anjan

**Affiliations:** 1 Miami Transplant Institute, Jackson Health System, Miami, FL 33136, USA; jxb1350@med.miami.edu (J.B.V.); gciancio@med.miami.edu (G.C.); gguerra@med.miami.edu (G.G.); labbo@med.miami.edu (L.A.); 2 Department of Medicine, Division of Infectious Disease, University of Miami Miller School of Medicine, Miami, FL 33136, USA; 3 Louis Calder Memorial Library, University of Miami Miller School of Medicine, Miami, FL 33136, USA

**Keywords:** *Nocardia*, nocardiosis, opportunistic infection, solid organ transplant, case series

## Abstract

Solid organ transplant recipients (SOTRs) are at an increased risk of nocardiosis, a rare but life-threatening opportunistic infection. Universal PCP prophylaxis with trimethoprim–sulfamethoxazole (TMP-SMX) is used at our center, which is active in vitro against most species of the *Nocardia* genus and may have a role in preventing early infections. This is a single-center retrospective cohort study of nocardiosis in adult SOTRs at a large transplant center between January 2012 and June 2022, with comprehensive review of literature. Out of 6179 consecutive cases, 13 (0.2%) were diagnosed with nocardiosis. The patients were predominantly male (76.9%) and kidney transplant recipients (62%). Infection was diagnosed at median of 8.8 months (range, 3.7–98) after transplant. Patients were followed for a median of 457 days (range 8–3367). Overall mortality within one year after diagnosis was 46% (6/13), of which 17% (1/6) of deaths was attributable to *Nocardia* infection. No recurrence was reported. *Nocardia* infections were noted in a small proportion of our SOTRs and carried significant morbidity and mortality. TMP-SMX prophylaxis may be protective in some cases given low incidence of cases.

## 1. Introduction

Nocardiosis is an opportunistic infection caused by *Nocardia* spp., a ubiquitous actinomycete found in decaying vegetation, soil, and fresh and salty water. As inhalation is the main form of entry for *Nocardia*, lung involvement is frequent, and bacteria may subsequently disseminate to distant areas, such as the skin/soft tissue, brain, or another vital organ [1].

Solid organ transplant recipients (SOTRs) are uniquely predisposed to nocardiosis due to their iatrogenic cell-mediated immune deficit necessary to avoid rejection and to maintain allograft function. The overall incidence of *Nocardia* infection in SOTRs ranges from 0.1 to 3.5%. Rates of infection vary based on the transplanted organ, with higher rates reported among thoracic transplant recipients [2,3,4,5,6,7]. Most cases of *Nocardia* infection occur at a median of 17.5 (range, 2–244) months after solid organ transplant (SOT) [4]. Early nocardiosis can occur in the setting of acute rejection treatment. 

The mainstay of *Nocardia* treatment is prolonged antibiotic therapy, although surgical debridement may also be required [8]. Despite treatment, the prognosis of nocardiosis in SOTRs is poor [9,10,11,12,13]. Here, we describe our experience at a tertiary transplant center.

## 2. Methods

### 2.1. Study Design and Patient Selection

This is a single-center, retrospective cohort of adult SOTRs with nocardiosis from 1 January 2012 to 30 June 2022 who underwent transplantation at the Miami Transplant Institute, Jackson Memorial Hospital (JMH), Miami, Florida, USA. Patient demographics, immunosuppressive regimen, use of PCP prophylaxis, rejection, concurrent opportunistic infections, clinical presentation, radiological findings, treatment, and outcomes were collected. 

### 2.2. Definition

*Nocardia* infection was defined as culture growth of *Nocardia* species with compatible signs, symptoms, and/or radiographic features. Sampling sites included blood, sputum, bronchoalveolar lavage (BAL), lung biopsy, skin biopsy, or abscess drainage of collections. Disseminated infection was defined as involvement of at least 2 non-contiguous sites, or isolated central nervous system (CNS) involvement. Determination of infection cure or nocardiosis as the attributable cause of death was performed by physician review of the medical record. 

Organisms were identified initially in the JMH microbiology laboratory using MALDI-TOF MS (Biomerieux, Marcy-l’Étoile, France). Specimens were then sent to the National Jewish Health Laboratories (Denver, CO, USA) for further identification by molecular methods if needed, and antimicrobial susceptibility testing. The institutional review board approved the study.

### 2.3. Statistical Analysis

Demographic data were analyzed using descriptive statistics. Categorical variables were reported as numbers and percentages. Continuous variables were reported as the median and interquartile range (IQR) or range.

### 2.4. Literature Review 

We conducted a comprehensive literature database search on Ovid Medline, Embase, CINAHL, and Cochrane CENTRAL to find all relevant literature, published from 1 March 1993 through 1 March 2023. We limited the search to studies on adult humans, in English. We included all randomized controlled trials (RCTs), cohort studies, and case series that included targeted data on *Nocardia* infection and SOT. We excluded the publications involving population other than SOTRs and case series with less than five SOTRs. Reports where information could not be extracted in detail were excluded. We initially found 616 citations. We used Covidence web-based software (www.covidence.org, accessed on 12 March 2023) to remove duplicates, screen titles and abstracts, and read full articles. In total, 17 publications met the criteria, including 13 retrospective reviews, 2 matched case–control studies, and 2 RCTs with a total of 475 patients (Table 1). Causative organisms included the following: *N. farcinica* (103/475; 21.6%), *N. asteroides* (90/475; 18.9%), *N. nova* (87/475; 16.8%), other *N. species* (82/475; 17.2%), *N. cyriacigeorgica* (42/475; 8.8%), *N. abscessus* (18/475; 3.7%), *N. brasiliensis* (15/475; 3.2%), *N. beijingensis* (11/475; 2.3%), *N. veterana* (7/475; 1.5%), *N. pseudobrasiliensis* (5/475; 1.0%), *N. wallacei* (3/475; 0.6%), *N. otitidiscaviarum* (3/475; 0.6%), *N. tranvalencis* (3/475; 0.6%), *N. flavorosea* (2/475; 0.4%), *N. brevicatena* (2/475; 0.4%), *N. paucivorans* (1/475; 0.2%), and *N. asiatica* (1/475; 0.2%). Overall *Nocardia* incidence ranged between 0.4 and 45%. The median time from transplant to *Nocardia* diagnosis ranged between 0 and 244 months post-transplant. Median duration of therapy ranged between 3 and 12 months. The recurrence rate ranged between 0 and 11%. Overall mortality ranged between 8.3% and 77%, with death attributable to *Nocardia* between 0 and 40%.

## 3. Results

### 3.1. Patient Characteristics

Between 1 January 2012 and 30 June 2022, 6179 SOTs were performed at JMH, including 3845 kidney, 1421 liver, 122 pancreas, 227 kidney–pancreas, 230 heart, 159 lung, 6 heart–lung, and 169 intestine. Within the entire cohort, a total of 13 patients were diagnosed with nocardiosis including 7/13 kidney alone (54%), 4/13 heart (30%), 1/13 double lung (8%), and 1/13 combined heart–kidney transplant recipients (8%) (Table 2). The overall rate of infection by *Nocardia* was 0.2%, with the following distribution according to transplant type: kidney 0.18% (7/3845), heart 1.73% (4/230), lung 0.62% (1/159) and combined heart–kidney 16.6% (1/6). The patients were predominantly male (77%) and African American (77%), with a median age of 54 years (range 26–66). Only one patient had a history of chronic corticosteroid use prior to SOT due to sarcoidosis. 

The induction regimen varied according to the SOT type. Five out of thirteen patients were diagnosed with acute rejection at a median of 97 days (range: 59–149) before diagnosis of *Nocardia* infection and were treated with augmented immunosuppression as per institutional protocol. Most patients (10/13) received a T-cell-depleting antibody in the preceding 12 months as either part of the induction regimen or rejection treatment. Prednisone was part of the maintenance regimen at the time of *Nocardia* diagnosis in 12/13 patients (92%), with a median dose of 20 mg daily. An elevated calcineurin inhibitor level in the preceding month was present in only 1/13 patients (7.5%). 

Nine out of thirteen patients were diagnosed with nocardiosis while receiving PCP prophylaxis with trimethoprim–sulfamethoxazole (TMP-SMX). Among patients on TMP-SMX prophylaxis, data on *Nocardia* susceptibility were available for 7/9 patients, and all isolates were susceptible to TMP-SMX. Compliance with TMP-SMX could not be evaluated. In addition, 2/13 patients were receiving PCP prophylaxis with dapsone, and 2/13 were not receiving any PCP prophylaxis due to remote history of transplantation.

Six out of thirteen patients (46%) had a concomitant opportunistic infection, particularly cytomegalovirus, invasive aspergillosis, and non-tuberculous mycobacteria. 

### 3.2. Clinical and Radiographical Characteristics

Time from transplantation to nocardiosis diagnosis ranged from 3.7 to 98 months (median 8.8 months). Lungs were the sole site of infection in 55% of the patients (7/13). Radiographic findings included lung mass, pulmonary nodules, ground-glass opacities, cavitary lesions, and halo signs. Four out of thirteen patients (30%) had extrapulmonary involvement in addition to pneumonia, including skin abscesses, bacteremia, and brain lesions. Isolated skin infection was present in 1/13 patients (7.5%), and isolated CNS infection was observed in 1/13 patients (7.5%). Of the 12 patients with no neurological symptoms, 11 (91.6%) underwent routine brain computerized tomography or magnetic resonance imaging, and 3/11 (27%) were found to have *Nocardia* metastatic CNS infection. 

Most patients required invasive work-up for *Nocardia* diagnosis, including bronchoscopy (6/13, 46%), lung biopsy (2/13, 15%), and skin biopsy (2/13, 15%). Out of 13 cases, 2 (15%) were diagnosed by blood cultures, and 1 (7.5%) was diagnosed by the Karius test. The Karius test is a non-invasive blood test based on next-generation sequencing of microbial cell-free DNA, able to diagnose clinically relevant bacteria, DNA viruses, fungi, and parasites. BAL or tissue *Nocardia* polymerase chain reaction was not performed. *Nocardia* species included *N. cyriacigeorgica* in 23% (3/13), *N. asteroides* in 7.5% (1/13), *N. nova* in 7.5% (1/13), *N. farcinica* in 7.5% (1/13), *N. abscessus* in 7.5% (1/13), *N. carnea* in 7.5% (1/13), and *N. niwae* in 7.5% (1/13) of patients. In contrast to our review of literature documenting *N. farcinica* to be the predominant species in SOTRs, our cohort had *N. cyriacigeorgica* as the most common isolate. Species identification of 4/13 isolates was not available. Time from symptom initiation to nocardiosis diagnosis ranged from 7 to 90 days (median 22 days). 

### 3.3. Management

Antibiotic susceptibility testing was available for 11/13 isolates and is outlined in Table 2. All strains were susceptible to TMP-SMX, imipenem (IPM), and linezolid (LZD). Combination therapy was implemented in all patients. Intravenous therapy was the initial route in 11/13 cases (84%). The most common empiric regimen was IPM plus TMP-SMX in 5/13 cases (38%), followed by IPM plus LZD in 3/13 cases (23%). Among the patients with susceptibility available, 11/11 received at least one drug with in vitro activity against the *Nocardia* isolate during the first 2 weeks. Ten out of eleven patients (90%) received an association of two appropriate antibiotics during the first 2 weeks of treatment. The median duration of therapy was 12 months (range 6–19 months) among the patients who completed treatment. Side effects were reported in 6/13 cases (46%), with hyperkalemia due to TMP-SMX being the most common. Secondary prophylaxis was utilized in 7/8 patients (87.5%).

### 3.4. Outcomes

One-year all-cause mortality was 46% (6/13), with 1/6 (17%) deaths attributable to nocardiosis. *Nocardia* infection did not directly contribute to 5/6 deaths, as determined by physician review of the medical record. Five out of thirteen patients (38%) achieved cure, with no recurrence after a median follow-up of 47 months (range 13.5–112.2 months).

## 4. Discussion

*Nocardia* infection was documented in 0.2% (13 of 6179) of our patients. The highest frequency corresponded to combined heart–kidney, followed by heart, lung, and kidney transplantation. Even though the overall rate of nocardiosis was lower than previously reported [2,3,6], it maintained the trend of higher rates among thoracic compared to abdominal transplant recipients. In our center, universal *PCP* prophylaxis with TMP-SMX is provided for a minimum of one year for non-lung transplant and lifelong for lung transplant recipients and is also active in vitro against most *Nocardia* species [5]. Our institutional prophylaxis protocol recommends one TMP-SMX double strength (DS) tablet Monday/Wednesday/Friday (MWF) or one TMP-SMX SS tablet daily for one-year post-transplant for heart, liver, kidney, pancreas, intestinal, and multi-visceral transplant and lifelong for lung transplant recipients. Dapsone or atovaquone are reserved for patients with documented sulfa allergy. Even though the data on the use of TMP-SMX prophylaxis for *Nocardia* infection have been mixed [2,25], some studies indicate a partial protective effect related to TMP-SMX dose and frequency [4,24,26,27]. We hypothesize that the low incidence of *Nocardia* infections in our series could be related to the use of universal TMP-SMX prophylaxis for a minimum of 12 months in our center. A comprehensive summary of existing literature is outlined in Table 1.

Time from transplantation to *Nocardia* diagnosis in our cohort ranged from 3 months to 8 years (median 8.8 months), occurring earlier than in previous reports [4] but in accordance with our review of the literature. Acute rejection treatment and a high rate of T-cell-depleting antibody use in the preceding 12 months could have favored the early development of nocardiosis in our series.

Guidelines for the treatment of nocardiosis in SOTRs have been published elsewhere [8]. An optimal management protocol has not been well established for SOTRs. An initial empiric regimen is typically applied and subsequently tailored based on antimicrobial susceptibility testing. Combination therapy should be considered in severe and/or disseminated disease, and several months of therapy are usually required. Secondary prophylaxis and reinstitution of primary prophylaxis following acute rejection should be considered, even though the best TMP-SMX dose and frequency have not been determined [4,24,26,27]. In our cohort, the median duration of therapy in 8/13 patients who completed treatment was 12 months, which is in accordance with the guidelines [8]. Most patients (7/8) received secondary prophylaxis following treatment. No recurrences were reported after a prolonged follow-up period. Despite treatment, only 54 patients had a favorable outcome in our series. *Nocardia* caused 17% of deaths. 

This study has several limitations of note. Firstly, it was retrospective and observational and is thus subject to intrinsic sources of bias. Secondly, we had a low number of events, which limited our analysis. The data on the duration of follow-up for the entire transplant cohort are not available, and loss of follow-up following transplantation could have contributed to the low incidence of cases.

Additionally, 4/13 isolates (30%) had no species identification, and 2/13 (15%) had no antimicrobial susceptibility testing available. 

In conclusion, nocardiosis is a rare but life-threatening opportunistic infection in immunocompromised hosts. *Nocardia* infection can lead to dissemination and death if untreated. A high index of suspicion is necessary even in the setting of TMP-SMX prophylaxis in SOTRs. 

## Figures and Tables

**Table 1:** Literature review and summary of nocardiosis in solid organ transplant recipients in the past 30 years.

Publication/Study Period	Study Type	Number of Patients	*Nocardia* Incidence	Infection Onset after Transplant	Most Common *Nocardia* Species	Sites Involved	PCP Ppx with TMP-SMX at the Time of *Nocardia* Diagnosis	Initial Combination Therapy	Median Duration of Therapy	Outcome
Arduino et al., 1993 [14] 1980–1992	Retrospective review	9	Kidney: 9/1255 (0.7%)	586 days post-transplant, mean (range 32 to 1806 days)	*N. asteroides*: 7/9 (78%) *N. brasiliensis* 2/9 (22%)	Lung: 8/9 (88%) Skin: 2/9 (22%) Disseminated: 2/9 (22%)	N/A	3/9 (33%) patients	6 months	Death: 1/9 (11%) Cure: 8/9 (89%) Recurrence: 1/9 (11%)
Santamaria Saber et al., 1993 [15] 1968–1991	Retrospective review	9	Kidney: 9/20 (45%)	6/9 (66%) of patients developed infection within 6 months post-transplant	*N. asteroides* 5/9 (55.5%) *N. brasiliensis* 1/9 (11.2%) *N. species*: 3/9 (33.3%)	Lung: 6/9 (66%) Skin: 3/9 (33%) Disseminated: 5/9 (55%)	N/A	N/A	6 months	Death: 7/9 (77%) Cure: 2/9 (22.2%) Recurrence: 0
Nampoory et al., 1996 [16] 1989–1985	Retrospective review	6	Kidney: 6/513 (1.16%)	16.3 ± 17.7 months post-transplant, mean (range 3–54 months)	*N. asteroides*: 4/6 (66.8%) *N. otitidiscaviarum* 1/6 (16.6%) *N. farcinica* 1/6 (16.6%)	Lung: 3/6 (50%) Skin: 3/6 (50%) Disseminated: 2/6 (33%)	N/A	4/6 (66%) patients	6 months	Death 2/6 (33%), 1/6 (16.5%) attributable to *Nocardia* Cure: 4/6 (66%) Recurrence: 0
Roberts et al., 2000 [17] 1989–1998	Retrospective review	10	Overall: 10/540 (1.85%) Transplant types: Heart: N/A Lung: N/A Heart and lung: N/A	14.5 ± 13 months post-transplant, mean (range 0–38 months)	*N. nova*: 6/10 (60%) *N. asteroides*: 2/10 (20%) *N. farcinica*: 1/10 (10%) *N. species*: 1/10 (10%)	Lung: 10/10 (100%) Skin: N/A Disseminated: N/A	3/10 (30%) patients	N/A	N/A	Death: 7/10 (70%), 0 cases attributable to *Nocardia* Cure: 5/10 (50%) Recurrence: 0
Hussain et al., 2002 [18] 1991–2000	Retrospective review	10	Lung: 10/473 (2.1%)	31 months post-transplant, median (range 5.6 to 102.8 months)	*N. farcinica*: 3/10 (30%) *N. nova*: 3/10 (30%) *N. asteroides*: 3/10 (30%) *N. brasiliensis*: 1/10 (10%)	Lung: 9/10 (90%) Skin: 0 Disseminated: 1/10 (10%)	6/10 (60%) patients	9/10 (90%) patients	6 months	Death: 4/10 (40%), 3/10 (30%) attributable to *Nocardia* Cure: 6/10 (60%) Recurrence: 1/10 (10%)
John et al., 2002 [19] 1971–2000	Retrospective review	27	Kidney: 27/1968 (1.3%)	19 months post-transplant, mean (range: 3–89 months)	*N. asteroides*: 27/27 (100%)	N/A	3/27 (11%) patients	N/A	6 months	Death: 7/27 (25%), 3/27 (11%) attributable to *Nocardia* Cure: 20/27 (74%) Recurrence: 0
Queipo-Zaragoza et al., 2004 [20] 1980–N/A	Retrospective review	5	Kidney 5/1239 (0.4%)	47.8 months post-transplant, mean (range 1–148 months)	*N. asteroides*: 3/5 (60%) *N. brasiliensis*: 2/5 (40%)	Lung: 3/5 (60%) Disseminated: 1/5 (20%) Skin: 1/5 (20%)	N/A	3/5 (60%) patients	Between 3 and 12 months	Death: 2/5 (40%), 2/5 (40%) attributable to *Nocardia* Cure: 3/5 (60%) Recurrence: 0
Peleg et al., 2007 [2] 1995–2005	Matched case-control study, single center	35	Overall: 35/5126 (0.6%) Transplant types: Lung: 18/521 (3.5%) Heart: 10/392 (2.5%) Intestinal: 2/155 (1.3%) Kidney: 3/1717 (0.2%) Liver: 2/1840 (0.1%)	22/35 patients (63%) diagnosed within 1-year post-transplant. 5/35 (14%) diagnosed 15 years post-transplant	*N. nova*: 17/35 (48.5%) *N. farcinica*: 9/35 (25.5%) *N. asteroides*: 8/35 (23%) *N. brasiliensis*: 1/35 (3%)	Lung: 27/35 (77%) Skin: 1/35 (2.8%) Disseminated: 7/35 (20%)	24/35 (69%) patients	35/35 (100%) patients	6 months (range 3 weeks to 12 months)	Death: 5/35 (14%), 4/35 (11.4%) attributable to *Nocardia* Cure: 31/35 (89%) Recurrence: 0
Poonyagariyagorn et al., 2008 [21] 1991–2007	Retrospective review	11	Lung: 11/577 (1.9%)	14.3 months post-transplant, mean (range 1.5–39 months)	*N. asteroides*: 6/11 (55%) *N. nova*: 2/11 (18%) *N. farcinica*: 1/11 (9%) *N. species:* 2/11 (18%)	Lung: 11/11 (100%) Skin (2/11 (18%) Disseminated: 3/11 (27%)	6/11 (54%) patients	6/11 (54.5%) patients	N/A	Death: 2/11 (18%), all attributable to *Nocardia* Cure: 9/11 (82%) Recurrence: 1/11 (9%)
Tripodi et al., 2011 [22] 1989–2008	Retrospective review	12	Heart: 12/508 (2.36%)	3 months post-transplant, median (range 1–7 months)	*N. cyriacigeorgica*: 3/12 (25.1%) *N. brasiliensis*: 1/12 (8.3%) *N. nova*: 1/12 (8.3%) *N. abscessus*: 1/12 (8.3%) *N. farcinica* 1/12 (8.3%) *N. asiática*: 1/12 (8.3%) *N. asteroides* (1/12 (8.3%) *N species*: 3/12 (25.1%)	Lung: 11/12 (91%) Skin: 0 Disseminated: 1/12 (8.3%)	N/A	12/12 (100%) patients	3 months	Death: 1/12 (8.3%), 0 attributable to *Nocardia* Cure: 11/12 (91.6%) Recurrence: 0
Santos et al., 2011 [3] 1980–2010	Retrospective review	19	Overall: 19/4600 (0.41%) Transplant types: Lung: 7/392 (1.78%) Heart 4/612 (0.65%) Kidney: 5/1900 (0.26%) Liver: 3/1654 (0.18%)	Range from 1 month to 13 years post-transplant	*N. asteroides*: 9/19 (47.4%) *N. species*: 5/19 (26.3%) *N. brasiliensis*: 2/19 (10.5%) *N. brevicatena*: 2/19 (10.5%) *N. otitidiscaviarum*: 1/19 (5.3%)	Lung: 17/19 (89%) Skin: 5/19 (26%) Disseminated: 4/19 (21%)	N/A	19/19 (100%) patients	N/A	Death: 10/19 (52%), 3/19 (15%) attributable to *Nocardia* Cure: 9/19 (47%). Recurrence: 0
De La Cruz et al., 2015 [23] 2006–2012	Retrospective review	19	N/A	282 days post-transplant, mean (range 8–3114 days)	*N. nova*: 6/19 (31.6%) *N. asteroides*: 5/19 (26.5%) *N. farcinica*: 3/19 (15.7%) *N. brasiliensis*: 2/19 (10.5%) *N. species*: 2/19 (10.5%) *N. transvalensis*: 1/19 (5.2%)	Lung: 15/19 (78.9%) Skin: 0 Disseminated: 3/19 (15.8%) Mediastinum: 1/19 (5.2%)	13 (68.4%) patients	18/19 (94%) patients	N/A	Death: 6/18 (33.3%), 2/18 (11%) attributable to *Nocardia* Cure: 16/19 (84.2%) Recurrence: 0
Coussement et al., 2016 [4] 2000–2014	Multicenter, retrospective, case–control	117	N/A	17.5 months post-transplant, mean (range 2–244 months)	*N. farcinica*: 41/117 (35%) *N. nova:* 28/117 (24%) *N. cyriacigeorgica*: 8/117 (7%) *N. abscessus*: 7/117 (6%) *N. veterana*: 7/117 (6%) *N. beijingensis*: 6/117 (5%) *N. flavorosea*: 2/117 (2%) Other: 6/117 (5%) *N. species*: 12/117 (10%)	Lung: 101/117 (86.3%) Skin: 37/117 (31.6%) Disseminated: 50 (42.7%) Joint: 3/117 (2.5%)	21/117 (17.9%) patients	N/A	N/A	Death: 19/117 (16.2%) Cure: 98/117 (83.7%) Recurrence: 6/117 (5.12%)
Majeed et al., 2018 [6] 1997–2016	Retrospective review	54	Overall: 54/2038 (2.65%) Transplant types: Lung 9.28% Heart and lung 5.8% Heart 4.57% Kidney 1.13% Liver 0.45%	N/A	*N. farcinica*: 15/54 (28%) *N. cyriacigeorgia*: 13/54 (24%) *N. beijingensis* 5/54 (9.5%) *N. abscessus*: 4/54 (7%) *N. nova*: 4/54 (7%) *N. brasiliensis* 3/54 (5.5%) *N. paucivorans* 1/54 (2%) Other: 9/54 (17%)	Lung: 49/54 (90%) Skin: 3/54 (5.5%) Disseminated: 13/54 (24%)	24/54 (44%) patients	39/54 (72%) patients	Between 1 month and >24 months	Death: 9/54 (17%), 1/54 (2%) attributable to *Nocardia* Cure: 45/54 (83%) Recurrence: 1/54 (2%)
Matchett et al., 2019 [7] 1999–2009	Retrospective review	10	Kidney: 0.1%	12 months post-transplant, mean (range, 6–102 months)	*N. farcinica*: 4/10 (40%) *N. nova:* 4/10 (40%) *N. species*: 2/10 (20%)	Lung: 10/10 (100%) Skin: N/A Disseminated: 4/10 (40%)	5/10 (50%) patients	6/10 (60%) patients	N/A	Death: 6/10 (60%), 3/10 (30%) attributable to *Nocardia* Cure: 7/10 (70%) Recurrence: 0
Goodlet et al., 2020 [24] 2012–2018	Matched case–control	20	Lung: 20/586 (3.4%)	9.4 months post-transplant, mean (range 4.4–55.2, months)	*N. farcinica*: 7/20 (35%) *N. cyriacigeorgica*: 3/20 (15%) *N. wallacei*: 3/20 (15%) *N. transvalensis*: 2/20 (10%) *N. otitidiscaviarum*: 1/20 (5%) *N. species*: 4/20 (20%)	Lung: N/A Skin: N/A Disseminated: 6/20 (30%)	12/20 (60%) patients	12/20 (60%) patients	N/A	Death: 5/20 (25%), all cases attributable to *Nocardia* Cure: 15/20 (75%) Recurrence: 2/20 (10%)
Yetmar et al., 2023 [10] 2000–2020	Multicenter retrospective cohort	102	N/A	400 days post-transplant, median (IQR: 155, 1487)	*N. farcinica*: 17/102 (16.7%) *N. nova*: 16/102 (15.7%) *N. cyriacigeorgica*: 15/102 (14.7%) *N. asteroides*: 10/102 (9.8%) *N. abscessus*: 6/102 (5.8%) *N. pseudobrasiliensis* 5/102 (4.9%) Other: 23/102 (22.6%) *N. species*: 10/102 (9.8%)	Lung: 111/125 (88.8%) Skin: 26/125 (20.8%) Disseminated: 18/125 (14.4%)	34/125 (27.2%) patients	97/125 (77.6%) patients	N/A	Death: 21/125 (16.8%), 9/125 (7.2%) attributable to *Nocardia* Cure: 104/125 (83.2%) Recurrence: 0

N/A: not applicable; PCP: *Pneumocystis carinii* pneumonia; Ppx: prophylaxis; TMP-SMX: trimethoprim–sulfamethoxazole.

**Table 2:** Clinical characteristics and outcomes.

No.	Age/Sex	Graft	Time to Transplant (Days)	Induction	Immunosuppression at the Time of Diagnosis	*Nocardia* Species	Site Involved	PCP Prophylaxis	Rejection Last 6 Months	Susceptibilities	Treatment	Outcomes
1	60/M	Heart	232	ALZ, MP	PRED, MMF, Tac	*N. species*	Disseminated	TMP-SMX DS three times weekly	No	TMP-SMX, IPM, LZD, CRO, FEP, TOB, CLR	TMP-SMX + IPM 6 wk followed by CRO + CLR 9 mo	Cure
2	39/M	DLT	749	Data unavailable	PRED, Tac	*N. cryriacigeorgica*	Lung	TMP-SMX SS three times weekly	Yes	Data unavailable	TMP-SMX + IPM	Death attributable to nocardiosis
3	63/F	DDKT	333	ATG, MP, BAS	PRED, MPA, Tac	*N. cryriacigeorgica*	Lung	TMP-SMX DS three times weekly	No	TMP-SMX, IPM, LZD, CRO, AMK, TOB	TMP-SMX + CRO 1 mo, followed by CRO 1 mo, followed by TMP-SMX 6 mo	Death unrelated to nocardiosis
4	66/M	Heart	184	ATG, MP	PRED, MMF, Tac	*N. asteroides*	Lung	TMP-SMX DS three times weekly	Yes	TMP-SMX, IPM, LZD, AMK, MIN, CLR	IPM + CRO 2 mo followed by TMP-SMX + MIN 6 mo	Cure
5	26/M	Heart and kidney	232	ATG, MP	PRED, MMF, Tac	*N. species*	Disseminated	TMP-SMX DS three times weekly	Yes	TMP-SMX, IPM, LZD, AMK, AMC	IPM + LZD 6 wk followed by LZD + AMC	Death unrelated to nocardiosis
6	62/M	DDKT	2962	ATG, MP, DB	PRED, MPA, Tac	*N. cryriacigeorgica*	Lung	No	Yes	TMP-SMX IPM, LZD, CRO, FEP	TMP-SMX + IPM 3 mo followed by TMP-SMX + CRO 3 mo	Cure
7	47/M	DDKT	318	ATG, MP, BAS, RIX	PRED, MMF, Tac	*N. species*	Lung	TMP-SMX SS three times weekly	No	TMP-SMX, IPM, LZD, CRO, AMK, MIN, CLR, AMC	LZD + CRO + MIN 3 mo followed by LZD + MIN 9 mo	Death unrelated to nocardiosis
8	64/F	Heart	220	ATG, MP	PRED, MPA, Tac	*N. nova*	Disseminated	Dapsone	No	TMP-SMX, IPM, LZD, AMK, CLR	TMP-SMX + LZD + AZM 2 wk followed by CRO + LZD 2 mo, followed by LZD alone	Death unrelated to nocardiosis
9	38/F	Heart	148	ATG, MP	PRED, MMF, Tac	*N. farcinica*	Lung	TMP-SMX DS three times weekly	No	TMP-SMX, IPM, LZD, AMK, MIN, CIP	IPM + LZD 2 wk followed by LZD + AMK + MIN 2 wk, followed by TMP-SMX + MIN + CIP 18 mo	Cure
10	50/M	DDKT	748	ATG, MP, BAS	PRED, MMF, Tac	*N. abscessus*	Disseminated	No	No	TMP-SMX IPM, LZD, CRO, AMK, TOB, MIN, CLR, AMC, MXF	TMP-SMX + IPM 1 mo followed by LZD + CRO 3 mo followed by LZD alone	Alive, on treatment
11	54/M	DDKT	111	ATG, MP, BAS	MPA, Tac	*N. carnea*	Lung	TMP-SMX SS three times weekly	No	TMP-SMX, IPM, LZD, AMK, MIN, CLR, CIP	TMP-SMX + IPM 2 mo followed by MIN 16 mo	Cure
12	58/M	DDKT	266	ATG, MP, BAS	PRED, MMF, Tac	*N. species*	Skin	Dapsone	No	TMP-SMX, IPM, LZD, CRO, AMK, MIN, CLR	IPM + LZD 1 mo followed by LZD	Death unrelated to nocardiosis
13	37/M	DDKT	380	ATG, MP, BAS	PRED, MMF, Tac	*N. niwae*	Brain	TMP-SMX DS daily	Yes	Data unavailable	LZD + CRO 2 mo	Alive, on treatment

## Data Availability

The raw data supporting the conclusions of this article will be made available by the authors on request.

## Abbreviations

ALZ	alemtuzumab
AMC	amoxicillin–clavulanic acid
AMK	amikacin
ATG	thymoglobulin
AZA	azathioprine
AZM	azithromycin
BAL	bronchoalveolar lavage
BAS	basiliximab
CIP	ciprofloxacin
CLR	clarithromycin
CNS	central nervous system
CRO	ceftriaxone
DB	daclizumab
DDKT	deceased-donor kidney transplant
DLT	double lung transplant
ESRD	end-stage renal disease
CEP	cefepime
GGO	ground-glass opacity
IPA	invasive pulmonary aspergillosis
IPM	imipenem
JMH	Jackson Memorial Hospital
LZD	linezolid
MAC	mycobacterium avium complex
MIN	minocycline
MMF	mycophenolate mofetil
MP	methylprednisolone
MPA	mycophenolic acid
MXF	moxifloxacin
MWF	Monday–Wednesday–Friday
N/A	not applicable
NICM	non-ischemic cardiomyopathy
PCP	*Pneumocystis* pneumonia
RCT	randomized controlled trial
RIX	rituximab
SOT	solid organ transplant
SOTRs	solid organ transplant recipients
Tac	tacrolimus
TMP-SMX	trimethoprim–sulfamethoxazole
TOB	tobramycin
Wk	weeks
